# A new species of karst-dwelling freshwater crab of the genus *Chinapotamon* Dai & Naiyanetr, 1994 (Crustacea: Decapoda: Brachyura: Potamidae), from Guizhou, southwest China

**DOI:** 10.7717/peerj.5947

**Published:** 2018-11-22

**Authors:** Jie-xin Zou, Jun Bai, Xian-min Zhou

**Affiliations:** 1 Research Laboratory of Freshwater Crustacean Decapoda & Paragonimus, School of Basic Medical Sciences, Nanchang University, Nanchang, Jiangxi Province, People’s Republic of China; 2 Key Laboratory of Poyang Lake Environment and Resource Utilization, Ministry of Education, Nanchang University, Nanchang, Jiangxi Province, People’s Republic of China

**Keywords:** Freshwater crab, New species, *Chinapotamon*, Taxonomy

## Abstract

*Chinapotamon maolanense* sp. n. from Maolan National Nature Reserve, Guizhou, southwest China, is described. *C. maolanense* sp. n. has diagnostic features of *Chinapotamon*, such as a slender and sinuous male first gonopod, prominently convex carapace, and one-third ratio of frons to carapace width. This new species can be distinguished from congeners by the combination of the following characters: relatively slender subterminal segment of the first gonopods, nearly oval-shaped carapace, anterolateral margin cristate of carapace and an oval-shaped gap between the fingers of the male major chela. In addition, we used a 16S rRNA gene fragment to explore the relationship between *C. maolanense* sp. n. and *C. glabrum*, *C. depressum* and other freshwater crabs distributed in Guizhou; the results support the new species being assigned to *Chinapotamon* and clearly different from other species used in the analysis.

## Introduction

Karst is a unique topography formed through the dissolution of soluble rocks such as limestone, dolomite, and gypsum. It is characterized by abundant underground drainage systems with sinkholes and caves (Ruan et al., 2013). The southwestern region of China exhibits the largest continuous distribution of karst topography in the world. The unique topography, water system, vegetation, tropical–subtropical warm–warm humid monsoon climate, and other natural geographical features of this area have created a tropical–subtropical karst ecosystem, that is, both typical and unique. Studies have shown that the ecological environment and biota of the karst terrain in the southwest region of China are extremely fragile, and many plant and animal species are rare or endangered (Liu et al., 2014; Tao, Zhou & Shui, 2011). Hence, detailed and comprehensive scientific research on the biodiversity of this area is urgently required.

**Figure 1:**
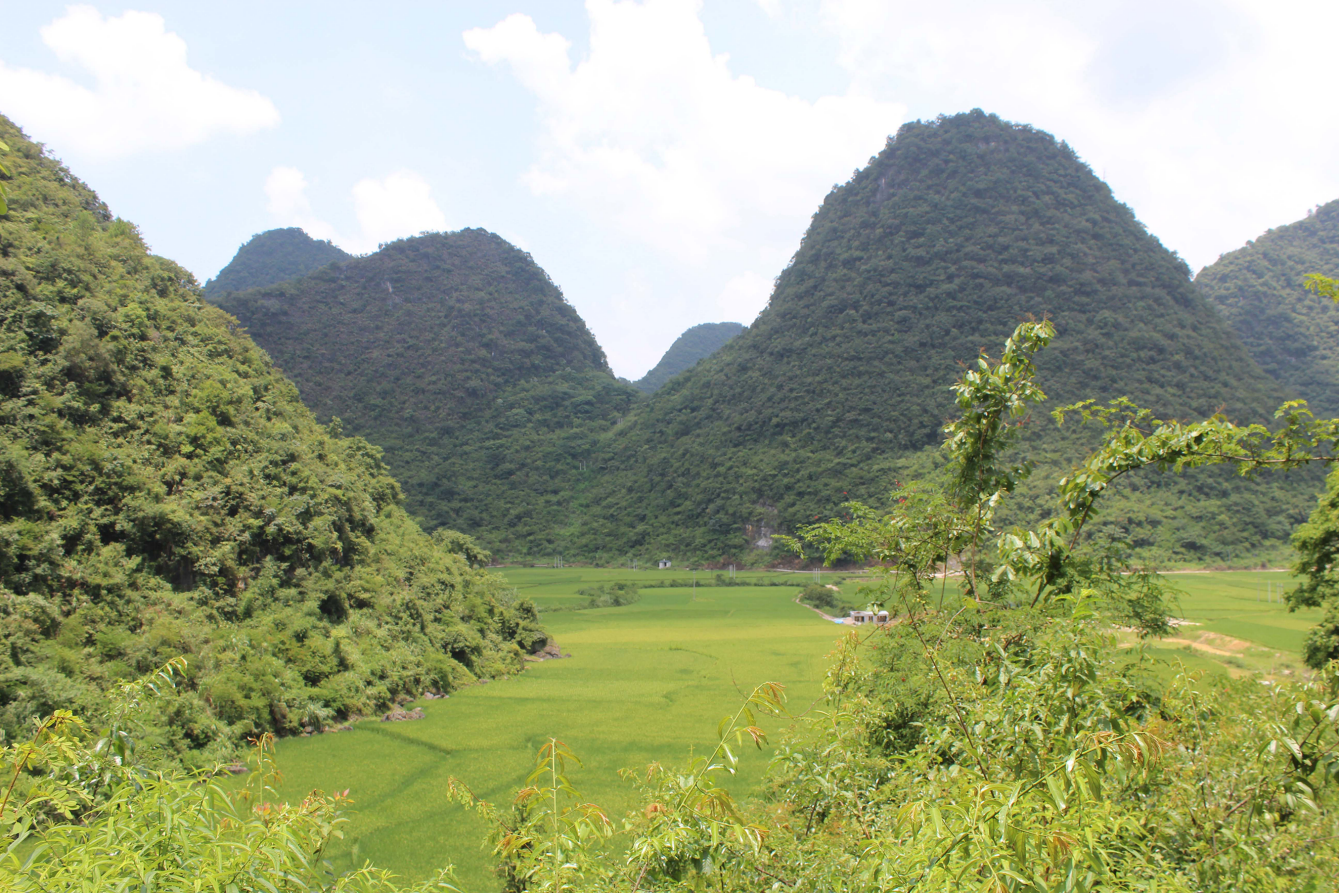
Typical karst terrain of Maolan National Nature Reserve. Photo taken by Xian-min Zhou, August, 2017.

The Maolan National Nature Reserve is located in Libo County, Guizhou Province, southwest China, in a subtropical monsoon humid climate zone. The region is characterized by a typical peak cluster funnel and peak cluster depression karst (Fig. 1), with numerous rare animals and plants. In the collection of freshwater crabs of the medical college of Nanchang University, Nanchang, we found an undescribed species of *Chinapotamon* (Dai & Naiyanetr, 1994) collected from the Maolan National Nature Reserve. Freshwater crabs of the genus *Chinapotamon*, 1994, include *Chinapotamon depressum* (Dai et al., 1980) (type species), *C. pusillum* (Song, 1984), *C. glabrum* (Dai et al., 1980), *C. longlinense* (Dai & Naiyanetr, 1994), *C. anlongense* (Dai & Naiyanetr, 1994), *C. xingrenense* (Dai & Naiyanetr, 1994), *C. dashiwei* (Ng, 2017), and *C. clarkei* (Ng, 2017). Thus far, *Chinapotamon* species have been found in Guizhou, Guangxi, and Guangdong Provinces of China. For comparison, holotypes of the previously described species deposited in the Institute of Zoology, Chinese Academy of Sciences (IZCAS), were examined, as were the descriptions of Dai (1999) and Ng (2017). In addition, mitochondrial 16S rRNA gene fragments were selected for phylogenetic analysis, and the genetic relationships among species are discussed.

## Materials and Methods

Specimens were collected from the Maolan National Nature Reserve in Guizhou, China; preserved in 95% ethanol; and deposited at the Department of Parasitology of the Medical College of Nanchang University (NCU MCP), Jiangxi, China. Sample collection was permitted by Authority of Maolan National Nature Reserve; the approval number is ML2010[49]. Carapace width and length were measured in millimeters. The abbreviations G1 and G2 are used for the male first and second gonopods, respectively. For comparison, specimens of congeners deposited at the IZCAS were examined, including *C. anlongense* (holotype, ♂, 23.26 × 30.85 mm) (CB05011), *C. depressum* (holotype, ♂, 28.32 × 36.78 mm) (CB05199), *C. glabrum* (holotype, ♂, 22.48 × 29.57 mm) (CB05198), *C. longlinense* (♂, 29.33 × 39.31 mm) (CB05201), and *C. pusillum* (holotype, ♂, 12.36 × 14.91 mm) (CB05188). The description, line drawing and images in Dai (1999) were also referenced; images in Ng (2017) were referenced for comparison with *C. dashiwei* and *C. clarkei*.

Approximately 50 mg of muscle tissue was excised from ambulatory legs and chelipeds. Total genomic DNA was extracted from the tissues using the DP1902 Tissue Kit (BioTeKe Inc., Beijing, China) following the manufacturer’s protocol. Then, an ∼550 base pair (bp) region of the 16S rRNA gene was amplified using polymerase chain reaction (PCR) with the primers 1471 (5′-CCTGTTTANCAAAAACAT-3′) and 1472 (5′-AGATAGAAACCAACCTGG-3′) (Shih, Ng & Chang, 2004). The PCR conditions were as follows: denaturation for 50 s at 94 °C, annealing for 40 s at 52 °C, and extension for 1 min at 72 °C (33 cycles), followed by a final extension for 10 min at 72 °C. The PCR products were purified and sequenced using an ABI 3730 automatic sequencer.

To further evaluate the validity and interspecies relationships of this new species, we performed molecular analysis with the mitochondrial 16S rRNA gene fragment. Fifteen species of 10 genera were downloaded from GenBank or sequenced, and *Portunus trituberculatus* was used as the outgroup (Table 1). Sequences were aligned using Muscle in MEGA 6.06 (Tamura et al., 2013), and the conserved regions were selected with Gblocks 0.91b (Castresana, 2000) using the default settings. Maximum likelihood (ML) analysis was conducted using the IQ-TREE web server (Trifinopoulos et al., 2016) with the default settings. MrBayes 3.2.6 (Ronquist & Huelsenbeck, 2003) was employed to perform Bayesian inference (BI) analysis, and four Monte Carlo Markov Chains of 2,000,000 generations were run with sampling every 1,000 generations. The first 500,000 generations were discarded as burn-in, and the consensus tree and Bayesian posterior probability were estimated using the remaining samples.

**Table 1:** The 16s gene sequences used in the molecular analysis.

Species	No./Accession No.	Locality	Author
*Chinapotamon maolanense*	11280060, 11280062	Wengdong Xiaoka, Baixian Hill, Banzhai Village, Lino County, Guizhou Province, China	This study
	11280063, 11280065		
	11280066, 11280067		
	11280008		
*Tenuilapotamon latilum bijiense*	11280072	Hongqi Natural Village, Wenchang Village, Luanchuan Town, Fengfeng County, Guizhou Province	This study
*Tenuilapotamon latilum anshunense*	11280078	Same as above	This study
*Longpotamon exiguum*	11280037, 11280041	Xiongjiapo Dongdong Bay, Fanxing Village, Nuxi Township, Jiangkou County, Guizhou Province	This study
*L. exiguum*	KT586114, KT586115	–	Ji et al. (2016)
*C. depressum*	KT586287	–	Ji et al. (2016)
*C. glabrum*	AB428451	–	Shih, Darren & Ng (2009)
*L. lansi*	KT586162, KT586163	–	Ji et al. (2016)
*Parapotamon spinescens*	AB428467	–	Shih, Darren & Ng (2009)
*Tiwaripotamon edostilus*	LC198523	–	Huang, Shih & Ng (2017)
*T. xiurenense*	LC198522	–	Huang, Shih & Ng (2017)
*Artopotamon latopeos*	MH045062	–	Chu, Wang & Sun (2018)
*Trichopotamon daliense*	AB428492	–	Shih, Darren & Ng (2009)
*Pararanguna semilunata*	AB428490	–	Shih, Darren & Ng (2009)
*Tenuipotamon huaningense*	AB428491	–	Shih, Darren & Ng (2009)
*Mediapotamon leishanense*	LC155164	–	Shih, Huang & Ng (2016)
*Portunus trituberculatus*	NC005037	–	Yamauchi, Miya & Nishida (2003)

The electronic version of this article in portable document format will represent a published work according to the International Commission on Zoological Nomenclature (ICZN), and hence the new names contained in the electronic version are effectively published under that Code from the electronic edition alone. This published work and the nomenclatural acts it contains have been registered in ZooBank, the online registration system for the ICZN. The ZooBank LSIDs (Life Science Identifiers) can be resolved and the associated information viewed through any standard web browser by appending the LSID to the prefix http://zoobank.org/. The LSID for this publication is: urn:lsid:zoobank.org:pub:96797C61-B883-4080-B157-4610D5D0CC14. The online version of this work is archived and available from the following digital repositories: PeerJ, PubMed Central, and CLOCKSS.

## Results

### Taxonomy

**Figure 2:**
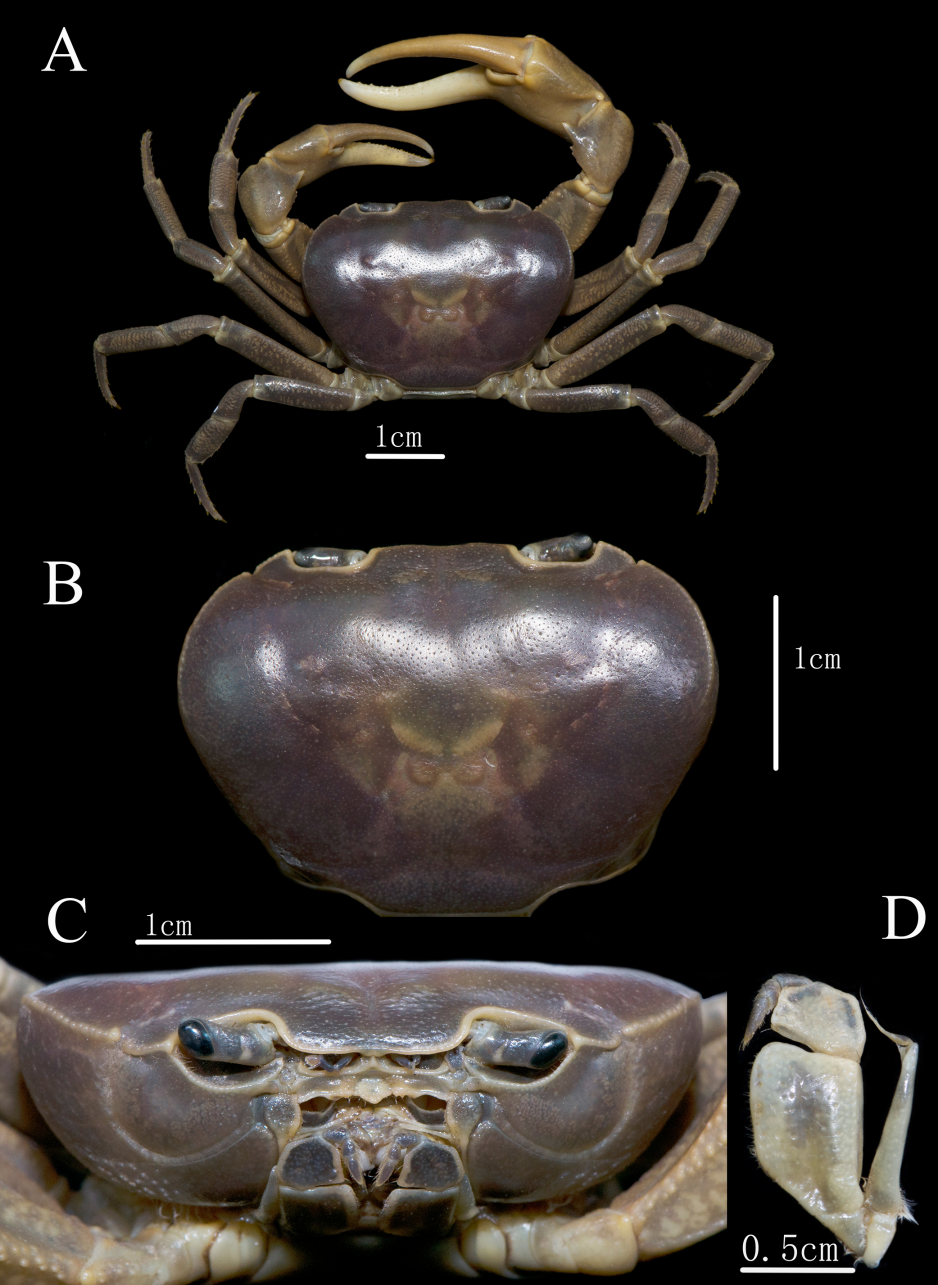
*C. maolanense* n. sp. Holotype male (35.8 × 25.9 mm) (NCU MCP 196101). (A) Overall habitus; (B) dorsal view of carapace; (C) frontal view of cephalothorax; (D) left third maxilliped. Photograph courtesy of Xian-min Zhou, August 2017.

**Figure 3:**
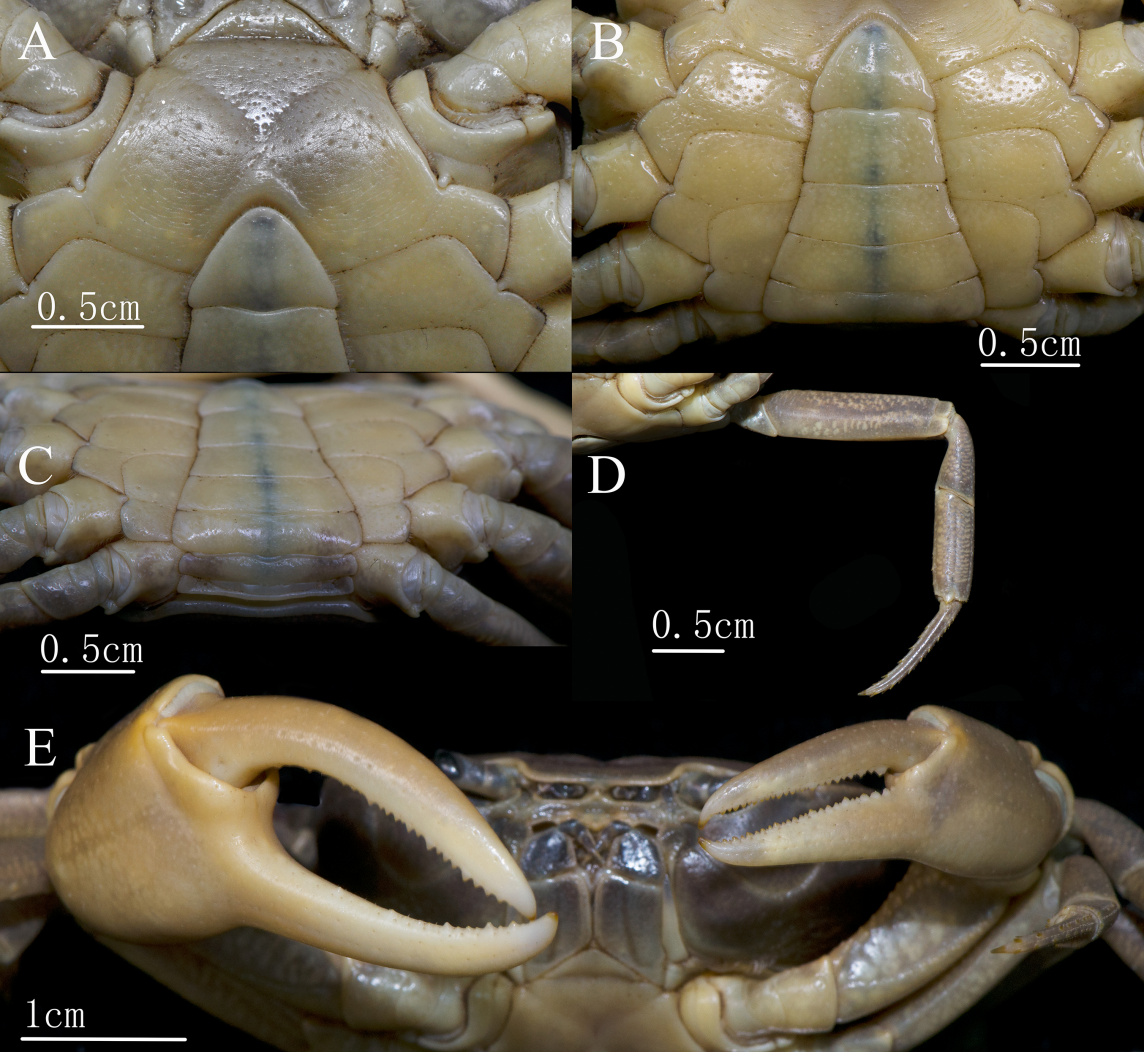
*C. maolanense* n. sp. Holotype male (35.8 × 25.9 mm) (NCU MCP 196101). (A and B) Anterior thoracic sternum, telson, and male pleonal somites 4–6; (C) posterior thoracic sternum, telson, and male pleonal somites 1–6; (D) right fourth ambulatory leg; (E) outer view of chelipeds. Photograph courtesy of Xian-min Zhou, August 2017.

**Figure 4:**
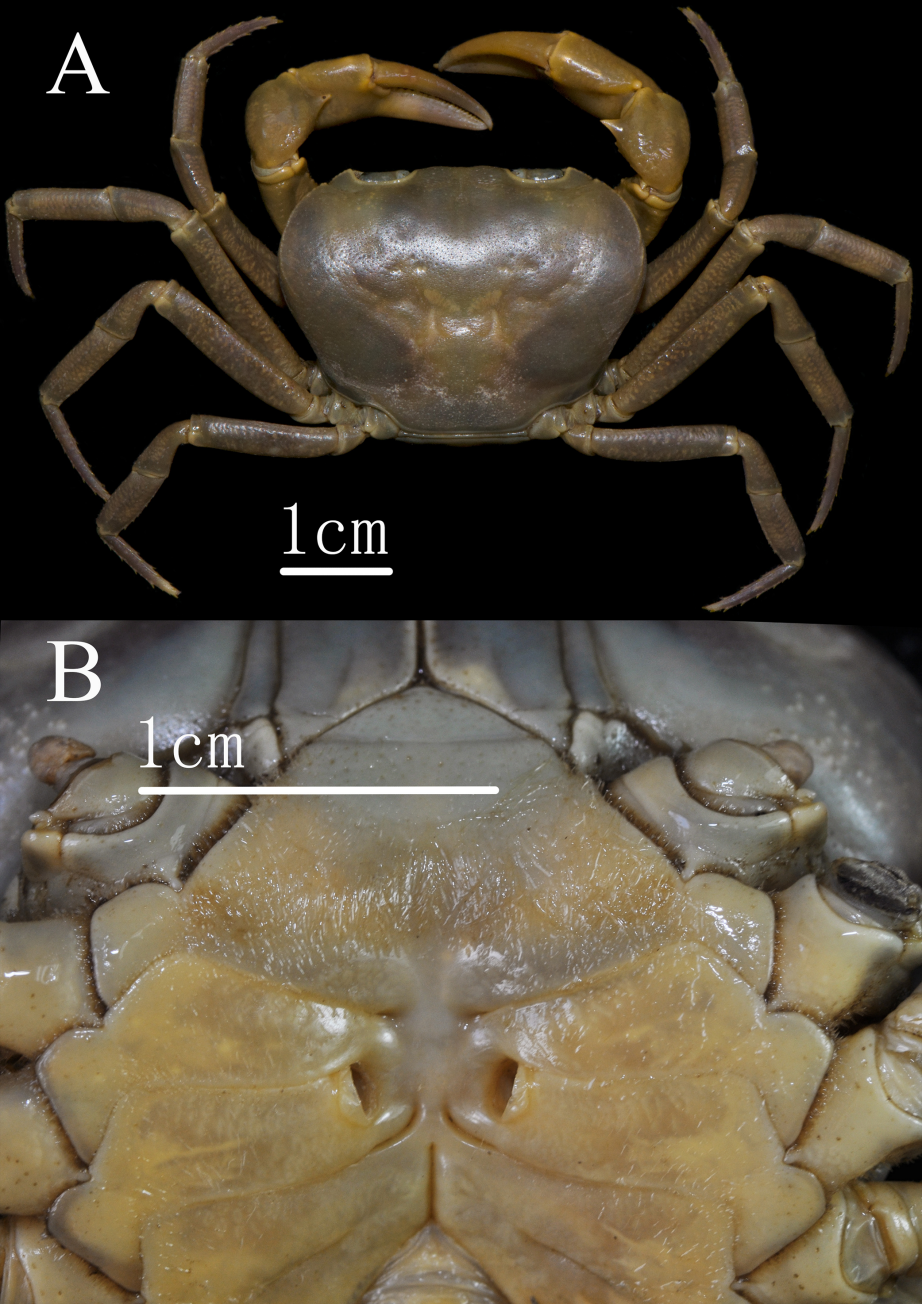
*C. maolanense* n. sp. Paratype female (36.7 × 26.6 mm) (NCU MCP 196107). (A) Overall habitus; (B) thoracic sternum showing vulvae. Photograph courtesy of Xian-min Zhou, August 2017.

**Figure 5:**
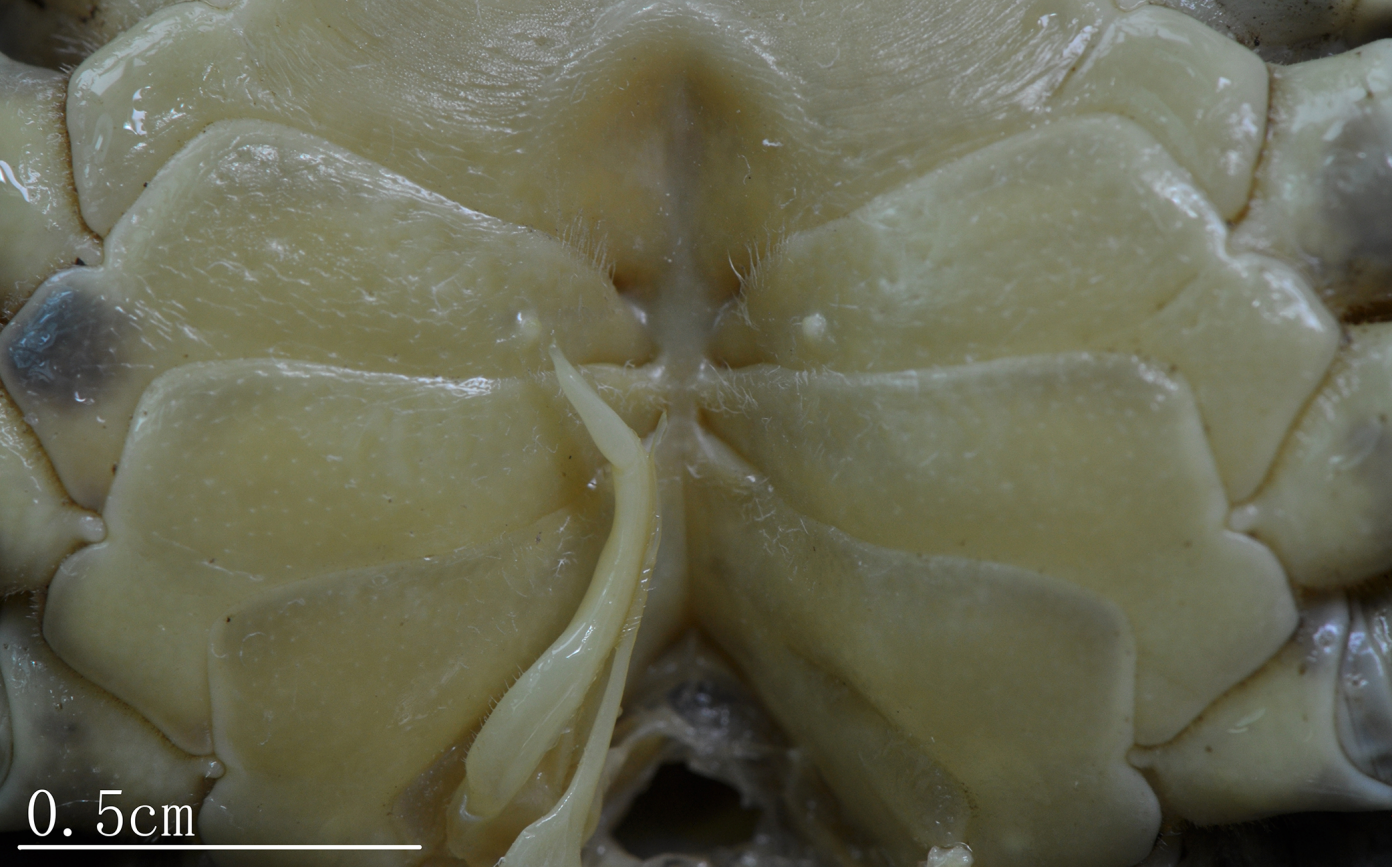
Natural position of male G1 and median longitudinal suture of sternites 7,8 *C. maolanense* n. sp. Paratype male (32.9 × 25.1 mm) (NCU MCP 196102). Photograph courtesy of Xian-min Zhou, August 2017.

**Figure 6:**
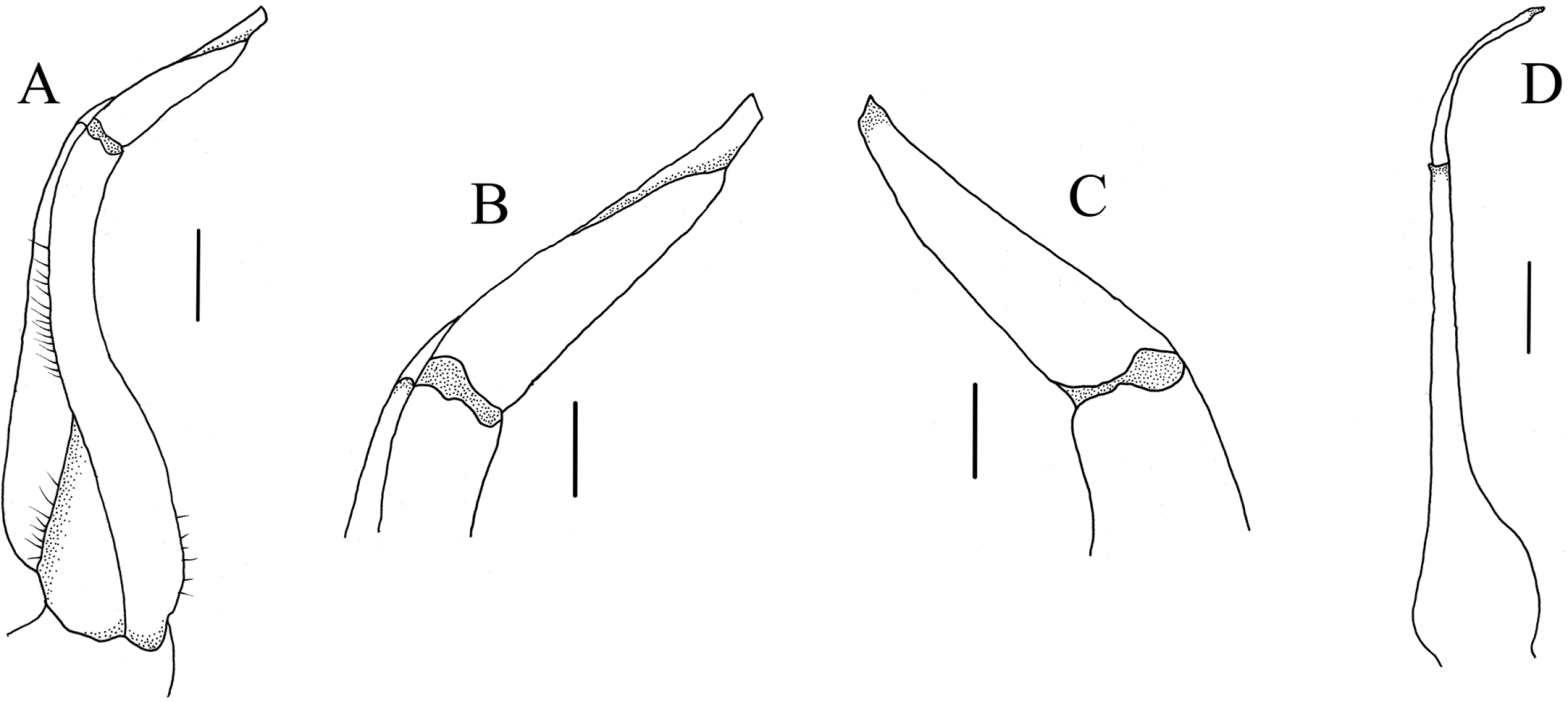
Gonopods. (A–D) *C. maolanense* n. sp. Holotype male (35.8 × 25.9 mm) (NCU MCP 196101); (A) ventral view of left G1; (B) ventral view of distal part of left G1; (C) dorsal view of distal part of left G1; (D) left G2. Scales: A, D = 1.0 mm; B, C = 0.5 mm.

**Figure 7:**
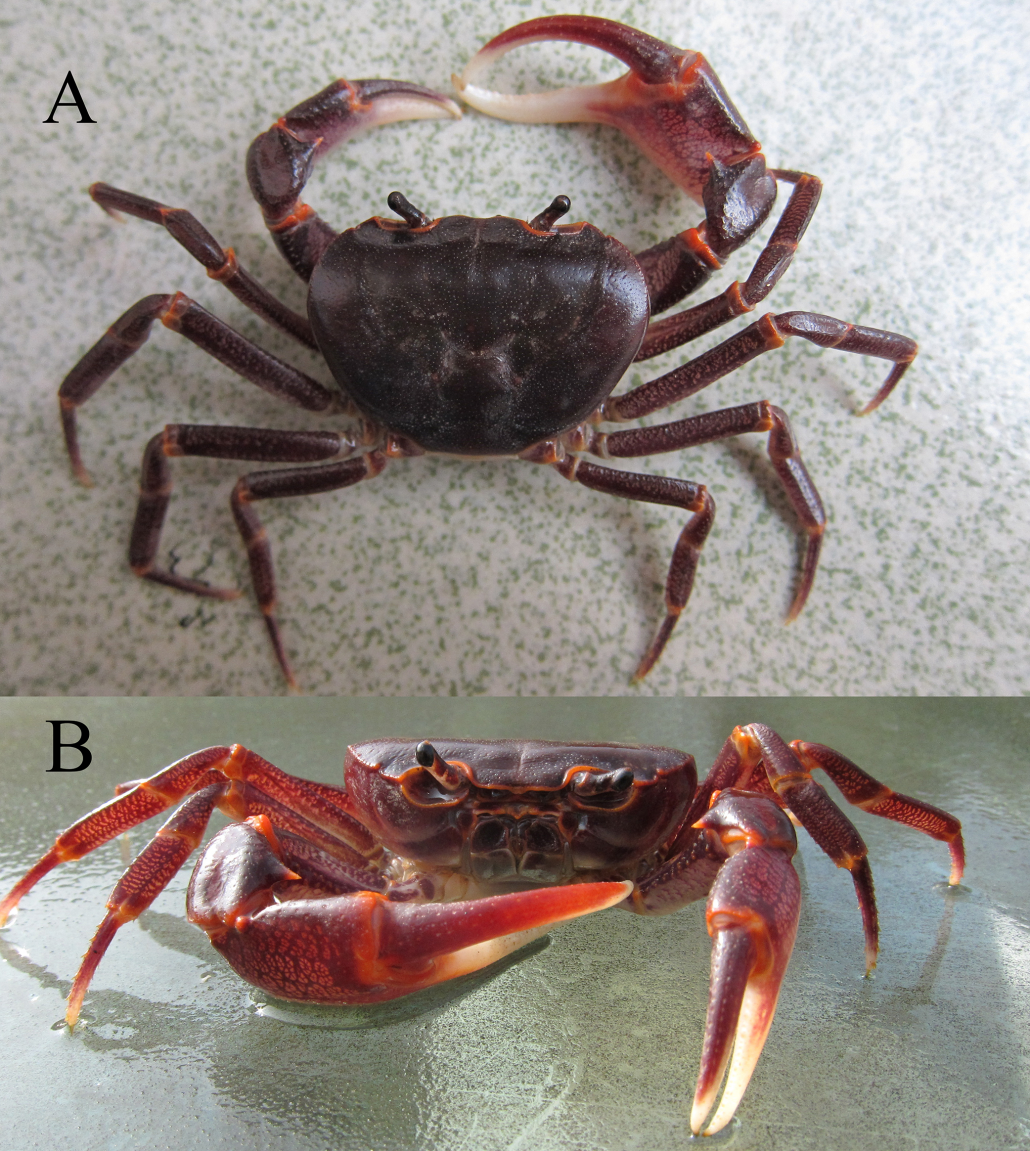
Living color. (A and B) *C. maolanense* n. sp. Paratype male (32.9 × 25.1 mm) (NCU MCP 196102). Photograph courtesy of Xian-min Zhou, October 2010.

**Family Potamidae Ortmann, 1896**
***Chinapotamon*Dai & Naiyanetr, 1994**
***Chinapotamon maolanense* sp. n. (Figs. 2–7)**

**Materials examined.** Holotype: ♂ (35.8 × 25.9 mm) (NCU MCP 196101), Wengdong Xiaoka, Baixian Hill, Banzhai Village, Lino County, Guizhou Province, 25.2344°N 108.0295°E, 532 m asl. Xian-min Zhou, October 2010. Paratypes: 1♀ (allotype) (36.7 × 26.6) (NCU MCP 196107), same data as holotype; 1♂ (32.9 × 25.1 mm) (NCU MCP 196102). Others: 5♂ (40.4 × 30.9, 30.2 × 22.8, 28.9 × 21.4, 27.8 × 20.7, 27.7 × 19.5) (NCU MCP 196103, 196105, 196106, 196109, 196110), same data as holotype; and 2♀ (38.5 × 29.7, 31.6 × 23.5) (NCU MCP 196104, 196108), same data as holotype.

**Description.** Carapace nearly oval, widest at anterior one-third, 1.3–1.4 times as broad as long (mean = 1.34, specimens in the **Materials examined** sections were measured); dorsal surface (Figs. 2A and 2B) smooth, with inconspicuous small granular depression, prominently convex horizontally at anterior one-third and bent forward and backward. Branchial regions slightly convex laterally, with inconspicuous granular depressions. Epigastric cristae low, separated by a narrow gap; central part of the epigastric region slightly depressed. Postorbital cristae very low, not fused with epigastric cristae. Anterolateral margin distinctly cristate, lined with granules. Frons approximately one-third as wide as the carapace. Orbits (Fig. 2C) suboval. Epistome longitudinally narrow, posterior margin with blunt median lobe (Fig. 2B). Ocular peduncle (Fig. 2C) relative slender, medially constricted, distal end (cornea) and base with approximately same diameter.

Ischium of third maxilliped (Figs. 2C and 2D) subtrapezoidal, approximately 1.4 times as long as broad (mid-length), with longitudinal depressions; merus trapezoidal, approximately 0.7 times as long as broad; exopod reaching proximal two-fifths of the merus, with slender flagellum exceeding the distal end of the merus when stretched anteriorly.

Male major chelipeds (Figs. 2A and 3E) unequal. Merus with inner-lower margin crenulated. Carpus surfaces smooth, with a long sharp inner angle, followed inferiorly by two small teeth at base; chela palm smooth in large males, approximately 1.2 times as long as broad; movable finger approximately 1.2 times as long as the immovable finger; both fingers slender, movable finger gradually curving downward, bending outward at base, immovable finger curving upward and outward at distal 2/3, forming a large oval gap, not crossing, occlusal margins of both fingers irregularly lined with short, sharp teeth distally. Minor chela shorter than major chela, and two fingers relatively straight, with a smaller gap when closed.

Ambulatory legs (Figs. 2A, 3D and 4A) long and slender. Outer surface of merus with shallow, irregular pits, length-to-width ratio of merus of the fourth leg 3.7; Surface of carpus with shallow, irregular pits, length-to-width ratio of the fourth leg 2.3; propodus subrectangular, with short sharp spines on the distal inner margins, length-to-width ratio of the fourth leg 3.0; dactylus gently curved, with sharp spines on both the inner and outer margins.

Male thoracic sternites (Figs. 3A, 3B and 5) 1 and 2 fused to form a triangular structure; sternites 2 and 3 demarcated by horizontal groove; sternites 3 and 4 fused, forming subtrapezoidal, superficially demarcated by oblique depressions; median longitudinal suture of sternites 7, 8 deep. Female vulvae (Fig. 4B) oval, deep, opening directed anteromesially.

Male pleon (Figs. 3A–3C) narrowly triangular; lateral margins of somites 3–6 forming slightly concave curvature; proximal margin of telson significantly wider than the distal margin of somite 6; somite 6 approximately 1.8 times as broad as long; telson approximately 1.3 times as broad as long.

G1 (Figs. 5 and 6A–6C) generally slender, sinuous, reaching or slightly not reaching the press button on thoracic sternite 5, but not reaching the suture between sternite 4/5; subterminal article directed inward proximally but slightly bent outward distally, approximately 2.2 times as long as the terminal article; terminal article tapering to subacute, slightly recurved tip. G2 (Fig. 6D) slightly shorter than G1, length of the basal segment approximately 2.7 times that of the flagelliform distal segment.

**Living color.** The dorsal surfaces of the carapace and pereopods are dark purple-red, and the joints of the cheliped merus and carpus and the ambulatory legs are bright red. The inner surface of the immovable finger and distal part of the movable finger are almost milky (Fig. 7).

**Etymology.** The species is named after the type locality: the Maolan National Nature Reserve.

**Figure 8:**
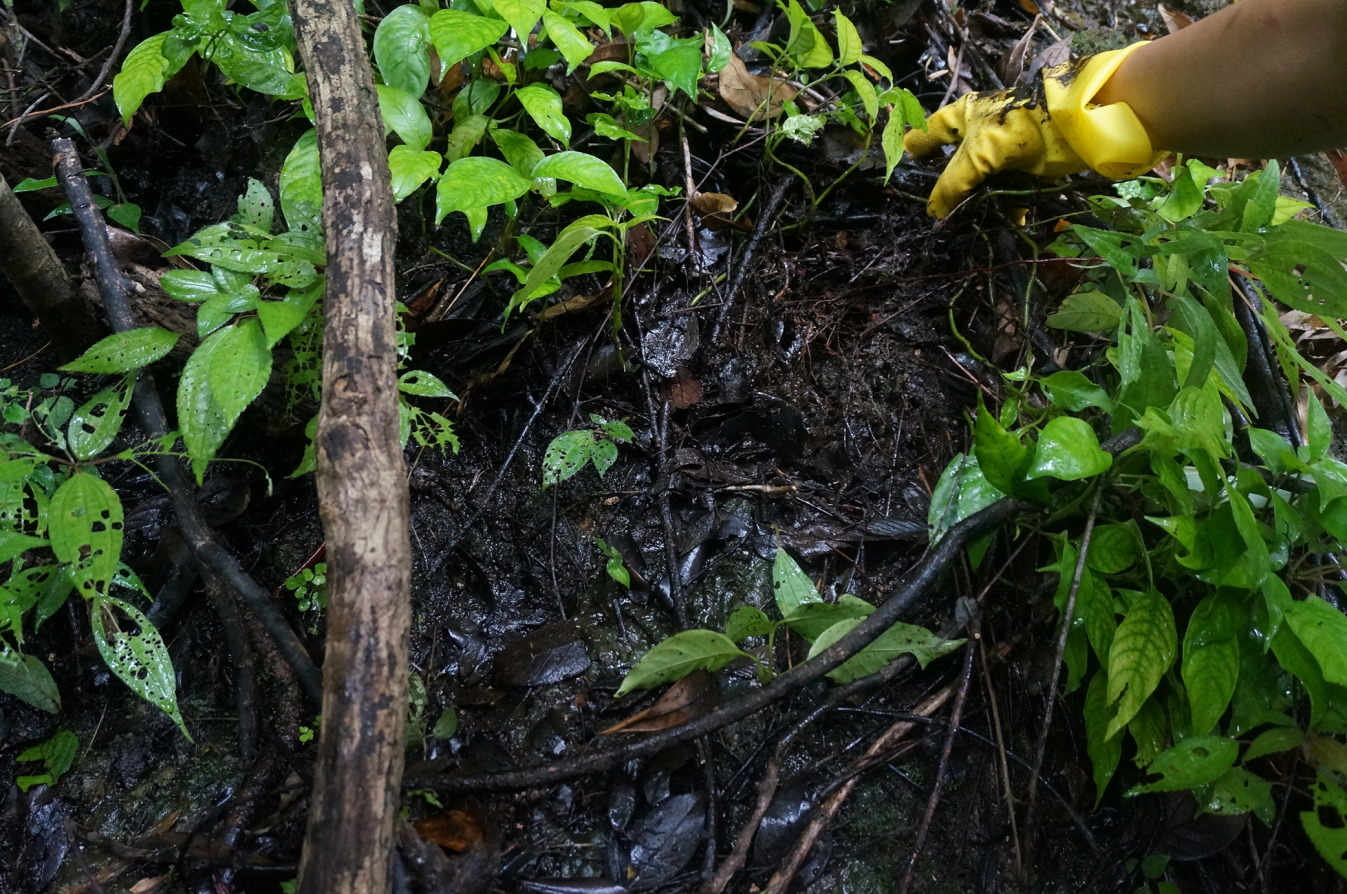
Habitat of *C. maolanense* sp. n. at the type locality. Photograph courtesy of Xian-min Zhou, October, 2010.

**Ecological note.** Karst terrain usually lacks soil and water, but in karst forests such as the Maolan National Nature Reserve, water could be conserved by dead branches and deciduous layers, and groundwater is another source. *C. maolanense* sp. n. crabs are locally known as “mountain crabs” because the species is generally distributed in low-altitude mountain forests. This species inhabits small mountain streams with low water flow and even moist soil where the surface has no flow; in contrast, most other *Chinapotamon* species live under stones in streams, while *C. dashiwei* and *C. clarkei* live in streams in caves. This species inhabits environments with dead leaves, dead branches, and humus (Fig. 8).

**Remarks.** *Chinapotamon* is characterized by the sinuous and slender G1, prominently convex dorsal surface of the carapace, and frons approximately one-third as wide as the carapace (Dai, 1999; Ng, 2017). *C. maolanense* sp. n. has all these features. Compared with the other *Chinapotamon* species, the shape of the G1 of *C. maolanense* sp. n. is similar to that of *C. anlongense*, *C. depressum*, *C. dashiwei*, and *C. clarkei*, but the terminal segment of the G1 of the new species is fairly straight, whereas those of the other four species are clearly curved. The subterminal segment of the G1 of *C. maolanense* sp. n. is almost as slender as the terminal segment, but the subterminal segment of the other four species are clearly stouter than the terminal segment. Additionally, the nearly ovate and prominent convex carapace, the large, oval-shaped gap between the fingers of the male major chela, and the uncountable granular teeth along the anterolateral margin of *C. maolanense* sp. n. are all distinguishable features (Table 2).

**Table 2:** Differences between *Chinapotamon* species.

	G1 and G2	Carapace dorsal surface	Male pleon	Male major chela	Fourth ambulatory leg	Eyes	Third maxilliped
*C. depressum*	G1 subterminal segment slender, terminal segment more slender, bent at about 30° outward. G2 basal segment to distal segment ratio 2.3.	Gently convex; anterolateral crista distinct, with uncountable granules; anterolateral region distinctly rugose; epigastric cristae distinct.	Male pleonal somite six broadly rectangular, width to length 1.8; telson width to length ratio 1.3.	Linear small gap when closed, with irregular relatively small serration on both fingers, palm 1.4 times as long as broad.	Merus length to width ratio 3.3, propodus ratio 1.7.	Peduncle long, eye filling orbit; cornea normal.	Ischium 1.5 times as long as broad.
*C. glabrum*	G1 subterminal segment slender, terminal segment more slender, bent at about 30° outward. G2 basal segment to distal segment ratio 2.2.	Convex; anterolateral crista low, with uncountable granules; anterolateral region smooth; epigastric cristae low.	Male pleonal somite six broadly rectangular, width to length 2.2; telson width to length ratio 1.4.	Linear small gap when closed, with irregular relatively small serration on both fingers, palm 1.3 times as long as broad.	Merus length to width ratio 3.6, propodus ratio 1.8.	Peduncle long, eye filling orbit; cornea normal.	Ischium 1.2 times as long as broad.
*C. anlongense*	G1 subterminal segment and terminal segment very slender, bent at about 20° outward. G2 basal segment to distal segment ratio 2.1.	Gently convex; anterolateral crista distinct, with 17–19 granules; anterolateral region gently rugose; epigastric cristae low.	Male pleonal somite six broadly rectangular, width to length 1.8; telson width to length ratio 1.3.	Linear small gap when closed, with irregular relatively small serration on both fingers, palm 1.3 times as long as broad.	Merus length to width ratio 3.1, propodus ratio 1.5.	Peduncle long, eye filling orbit; cornea normal.	Ischium 1.5 times as long as broad.
*C. xingrenense*	G1 subterminal segment stout, terminal segment more slender, bent at about 45° outward. G2 basal segment to distal segment ratio 2.1.	Gently convex; anterolateral crista distinct, with 17–19 granules; anterolateral region gently rugose; epigastric cristae low.	Male pleonal somite six broadly rectangular, width to length 1.9; telson width to length ratio 1.2.	Linear wide gap when closed, with obvious irregular serration on both fingers, basal tooth enlarged, palm 1.4 times as long as broad.	Merus length to width ratio 3.4, propodus ratio 1.6.	Peduncle long, eye filling orbit; cornea normal.	Ischium 1.5 times as long as broad.
*C. longlinense*	G1 subterminal segment stout, terminal segment more slender, bent at about 30° outward. G2 basal segment to distal segment ratio 2.2.	Gently convex; anterolateral crista distinct, with 15–18 granules; anterolateral region distinctly rugose; epigastric cristae low.	Male pleonal somite six broadly rectangular, width to length 1.6; telson width to length ratio 1.3.	Oblate wide gap when closed, with irregular serration on both fingers, basal tooth enlarged, palm 1.3 times as long as broad.	Merus length to width ratio not known, propodus ratio 1.6.	Peduncle long, eye filling orbit; cornea normal.	Ischium 1.5 times as long as broad.
*C. pusillum*	G1 subterminal segment slender, terminal segment more slender, directly upward. G2 basal segment to distal segment ratio 2.0.	Gently convex; anterolateral crista distinct, with uncountable denticle; anterolateral region distinctly rugose; epigastric cristae relatively distinct.	Male pleonal somite six broadly rectangular, width to length 2.0; telson width to length ratio 1.3.	Almost no gap when closed, with tiny serration on both fingers, palm 1.3 times as long as broad.	Merus length to width ratio 3.1, propodus ratio 1.6.	Peduncle long, eye filling orbit; cornea normal.	Ischium 1.6 times as long as broad.
*C. dashiwei*	G1 subterminal segment stout, terminal segment stouter, bent at about 30° outward. G2 basal segment to distal segment ratio 2.6.	Gently convex; anterolateral crista distinct; anterolateral region gently rugose; epigastric cristae low.	Male pleonal somite six broadly rectangular, width to length 2.0; telson width to length ratio 1.5.	Linear wide gap when closed, with obvious irregular serration on both fingers.	Merus length to width ratio 3.1, propodus ratio 2.2.	Peduncle long, eye filling orbit; cornea normal.	Ischium 1.4 times as long as broad.
*C. clarkei*	G1 subterminal segment stout, terminal segment more slender, bent at about 30° outward. G2 basal segment to distal segment ratio 2.6.	Gently convex; anterolateral crista distinct; anterolateral region gently rugose; epigastric cristae low.	Male pleonal somite six broadly rectangular, width to length 2.1; telson width to length ratio 1.4.	Oblate wide gap when closed, with obvious irregular serration on both fingers, basal tooth enlarged.	Merus length to width ratio 4.0, propodus ratio 2.4.	Peduncle shorted, eye not filling orbit; cornea reduced.	Ischium 1.3 times as long as broad.
*C. maolanense*	G1 subterminal segment and terminal segment very slender, bent at about 45°. G2 basal segment to distal segment ratio 2.7.	Prominent convex; anterolateral crista distinct, with unconspicuous uncountable granules; anterolateral region smooth; epigastric cristae low.	Male pleonal somite six broadly rectangular, width to length 1.8; telson width to length ratio 1.3.	Oval wide gap when closed, with tiny serration on distal part of both fingers, palm 1.2 times as long as broad.	Merus length to width ratio 3.7, propodus ratio 3.0.	Peduncle long, eye filling orbit; cornea normal.	Ischium 1.4 times as long as broad.

**Note:**

Modified from Ng (2017).

### DNA analysis

In this research, 25 16S rRNA sequences of 461 bp were obtained for subsequent analysis, including 15 species in 10 genera, most of which are distributed in southwestern China. The pairwise distance based on the K-2-P model showed that most of the pairwise genetic distances between the 15 species reached a threshold of 0.02 (Table 3); some were slightly below this value. The genetic distances between *C. maolanense* sp. n. and the other two *Chinapotamon* species ranged from 0.027 to 0.040. The minimum value between *Chinapotamon* species and the other genera was 0.095, supporting the new species being assigned to *Chinapotamon* but still maintaining species-level differences.

**Table 3:** Mean genetic distance among the 15 species based on the K-2-P model.

	①	②	③	④	⑤	⑥	⑦	⑧	⑨	⑩	⑪	⑫	⑬	⑭
① *Chinapotamon maolan*														
② *C. glabrum*	0.027													
③ *C. depressum*	0.040	0.018												
④ *Tenuilapotamon latilum bijiense*	0.124	0.116	0.108											
⑤ *Tenuilapotamon latilum anshunense*	0.124	0.116	0.108	0.000										
⑥ *Longpotamon exiguum*	0.106	0.102	0.106	0.037	0.037									
⑦ *L. lansi*	0.108	0.106	0.109	0.043	0.043	0.014								
⑧ *Parapotamon spinescens*	0.106	0.098	0.095	0.055	0.055	0.053	0.050							
⑨ *Tiwaripotamon edostilus*	0.123	0.120	0.118	0.113	0.113	0.106	0.097	0.083						
⑩ *Tiwaripotamon xiurenense*	0.134	0.121	0.123	0.100	0.100	0.096	0.093	0.088	0.036					
⑪ *Artopotamon latopeos*	0.155	0.152	0.158	0.113	0.113	0.114	0.105	0.100	0.141	0.141				
⑫ *Trichopotamon daliense*	0.160	0.158	0.169	0.118	0.118	0.117	0.110	0.110	0.149	0.149	0.031			
⑬ *Pararanguna semilunata*	0.157	0.149	0.155	0.118	0.118	0.115	0.108	0.102	0.143	0.143	0.027	0.036		
⑭ *Tenuipotamon huaningense*	0.166	0.160	0.163	0.121	0.121	0.121	0.113	0.105	0.141	0.136	0.038	0.054	0.052	
⑮ *Mediapotamon leishanense*	0.124	0.116	0.124	0.034	0.034	0.033	0.040	0.064	0.105	0.095	0.118	0.118	0.120	0.118

**Figure 9:**
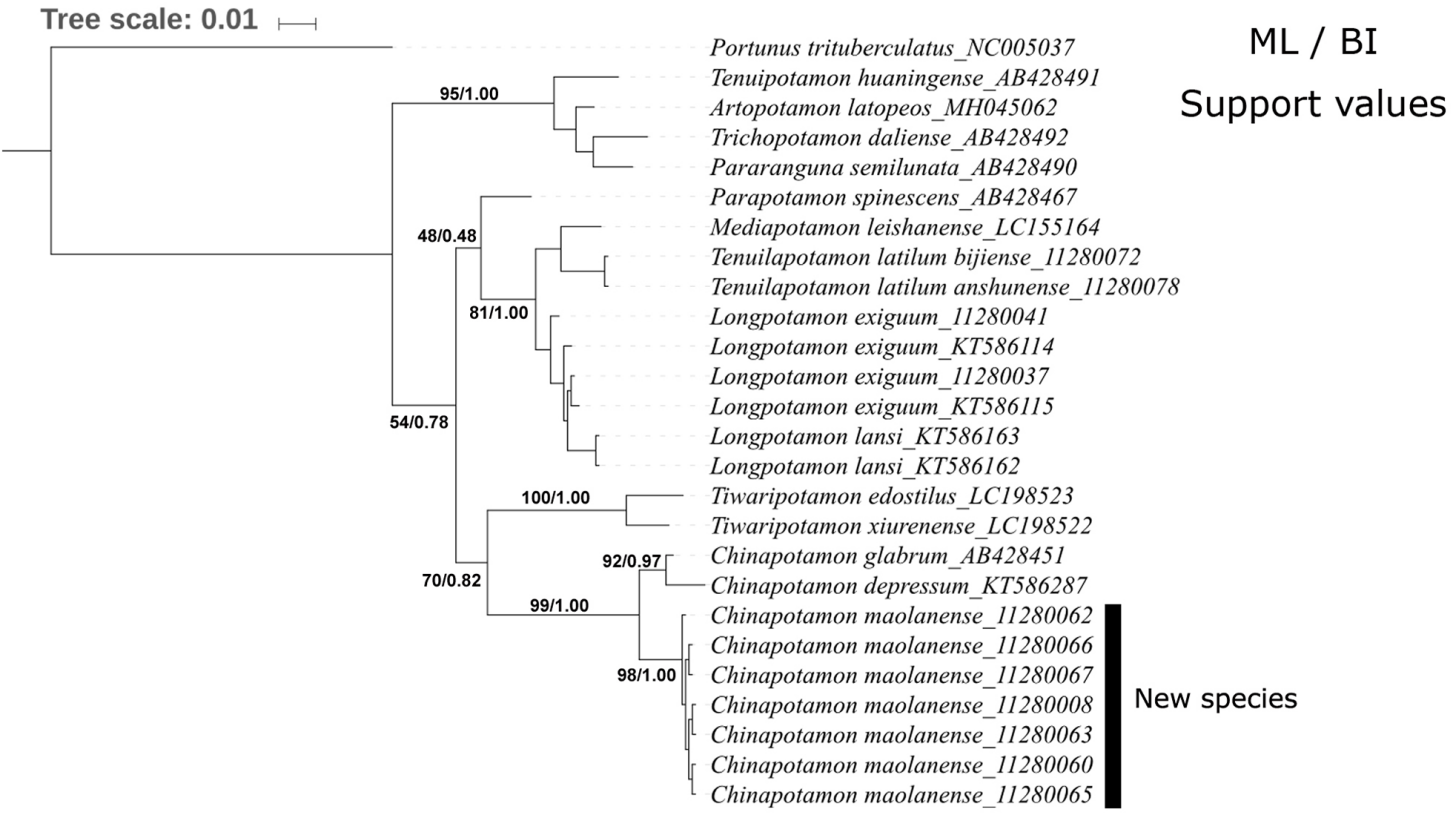
Phylogenetic tree for 15 species of freshwater crabs based on 16S rDNA.

The phylogenetic trees constructed by the BI and ML methods showed a similar topology, with credible support values (Fig. 9). The phylogenetic trees strongly supported the monophyly of *Chinapotamon* species used in the analysis. The seven specimens of *C. maolanense* sp. n. exhibited minimal intraspecific genetic variation; *C. depressum* and *C. glabrum* form a sister clade to *C. maolanense* sp. n. *Chinapotamon* is the next sister to the genus *Tiwaripotamon,* which is mostly distributed in Guangxi Province, unlike the other genera in Guizhou Province. *Tenuilapotamon, Longpotamon*, *Parapotamon*, and *Mediapotamon* form another clade sister to *Chinapotamon* and *Tiwaripotamon*, *Tenuipotamon*, *Artopotamon*, *Trichopotamon*, and *Pararanguna* form a clade at the base. This result indicates that there are at least three lineages of freshwater crabs in the mountainous terrain of southwestern China, and they may have different origins.

## General Discussion

China has the largest area of karst in the world, most of which is located in the subtropical climate zone in southern China (Liu et al., 2014; Zhou, 1987). Based on theoretical inference or speculation due to existing forest fragments, these karst landforms are believed to have been covered with dense forest vegetation before human influence, but these forests are gradually disappearing and are nearly destroyed (Han, Tang & Wu, 2010). Thus, the discovery of the Maolan karst forest and the primitiveness and richness of the forest vegetation in this area have attracted attention from researchers (Han, Tang & Wu, 2010), but biodiversity surveys in this region have mainly focused on plants (Zhou, 1987). Among the collections of freshwater crab specimens in this area, we found a new species described herein as *C. maolanense* sp. n.

Most known *Chinapotamon* species are distributed in Guangxi and Guangdong Provinces, China, except for *C. anlongense* and *C. xingrenense* from southwest Guizhou Province, where the average altitude is 1,000–2,000 m. Dai (1999) also collected observations at altitudes above 1,000 m. However, the average altitude of the type locality of *C. maolanense* in southern Guizhou Province is approximately 500 m, and the main terrain of this area is a low karst peak cluster instead of high mountains. In contrast to the other karst-dwelling crabs, the specimens of *C. maolanense* were not collected from caves but rather from the humus layer in the forest with small water flow or even just a wet environment; this is a rare habitat for freshwater crabs. In this case, we speculate that this species may have more athletic ability.

## Supplemental Information

10.7717/peerj.5947/supp-1 Supplemental Information 1 BI tree.

10.7717/peerj.5947/supp-2 Supplemental Information 2 ML tree.

10.7717/peerj.5947/supp-3 Supplemental Information 3 16s rRNA sequences.

## Additional Information and Declarations

### Field Study Permissions

The following information was supplied relating to field study approvals (i.e., approving body and any reference numbers): Sample collection was permitted by the Authority of Maolan National Nature Reserve (approval number ML2010[49]).

### Data Availability

The following information was supplied regarding data availability: The DNA sequences and phylogenetic trees are provided in the Supplemental Files.

### New Species Registration

The following information was supplied regarding the registration of a newly described species: Publication LSID: urn:lsid:zoobank.org:pub:96797C61-B883-4080-B157-4610D5D0CC14. *Chinapotamon maolanense* LSID: urn:lsid:zoobank.org:act:6245872A-6394-4815-B61D-07CAE7B7A624.
